# Absent Contrast Filling of Ipsilateral Superficial Middle Cerebral Vein Predicts Midline Shift in Acute Middle Cerebral Artery Occlusion

**DOI:** 10.3389/fneur.2020.570844

**Published:** 2020-11-05

**Authors:** Sheng Zhang, Longting Lin, Ruiting Zhang, Meiping Wang, Yannan Yu, Zongjie Shi, Mark Parsons, Yu Geng

**Affiliations:** ^1^Department of Neurology, People's Hospital of Hangzhou Medical College, Zhejiang Provincial People's Hospital, Hangzhou, China; ^2^Department of Neurology, John Hunter Hospital, University of Newcastle, Hunter Medical Research Institute, Newcastle, NSW, Australia; ^3^Department of Radiology, The Second Affiliated Hospital of Zhejiang University, School of Medicine, Hangzhou, China; ^4^School of Medicine, Stanford University, Los Angeles, CA, United States; ^5^Department of Neurology, Melbourne Brain Centre, The Royal Melbourne Hospital, University of Melbourne, Melbourne, VIC, Australia

**Keywords:** superficial middle cerebral vein, ischemic core, midline shift, reperfusion, computed tomography perfusion

## Abstract

**Background and purpose:** Midline shift is a life-threatening complication of acute large artery occlusion (LAO). The value of superficial middle cerebral vein (SMCV) for predicting midline shift is currently unclear for patients with acute LAO.

**Methods:** Consecutive acute LAO (middle cerebral artery M1 ± intracranial internal carotid artery) patients between March 2018 and May 2019 were included. Absent filling of ipsilateral cortical vein (marked as SMCV–) was defined as no contrast filling into the vein across the whole venous phase of four-dimensional computed tomography (CT) angiography derived from CT perfusion in the ischemic hemisphere.

**Results:** In the total of 81 patients, 31 (38.4%) were identified as SMCV–. SMCV– independently predicted midline shift, with sensitivity of 87.5% and specificity of 82.5%. Receiver operating characteristic analysis showed that including SMCV– as a predictor in addition to baseline ischemic core volume significantly increased the area under the curve in predicting midline shift (SMCV– with baseline ischemic core volume vs. baseline ischemic core volume: AUC = 0.903 vs. 0.841, *Z* = 2.451, *P* = 0.014).

**Conclusion:** In acute LAO patients, the presence of SMCV– was a sensitive and specific imaging marker for midline shift. SMCV– had supplementary value to baseline ischemic core volume in predicting midline shift.

## Introduction

Midline shift caused by edema is a life-threatening complication of acute large artery occlusion (LAO) (1). Treatment options for midline shift are still limited. Only decompressive craniectomy performed within 48 h of stroke onset was shown to reduce mortality, particularly when undertaken before signs of herniation appear (2). Therefore, early detection of midline shift is very important.

Large ischemic core has been known as the key risk factor for midline shift, as the severity of ischemia is associated with the degree of cytotoxic edema (3, 4). In recent years, impairment of venous drainage was also found to play a pivotal role in the development of cerebral edema. The superficial middle cerebral vein (SMCV) is a large superficial cerebral vein that can be easily identified through angiography. It has been reported that the filling deficit of SMCV was associated with cerebral edema after clipping surgery of intracranial aneurysms (5). The advances in four-dimensional computed tomography (CT) angiography (4D-CTA) derived from CT perfusion (CTP) imaging allow the noninvasive examination of cerebral veins. We have previously found that the absent filling of SMCV on 4D-CTA was associated with cerebral edema growth and poor functional outcome in stroke patients who received thrombolysis (6), demonstrating that SMCV could serve as a sensitive marker for predicting edema. However, the association of SMCV with midline shift in acute LAO patients remains unclear.

Therefore, in this study, we aimed to explore the effect of the absent filling in SMCV on 4D-CTA on the development of midline shift in acute LAO patients and compare its predictive value with that of ischemic core volume.

## Methods

### Ethics Statement

All patients or appropriate family members had given written informed consent prior to the study. The protocols of the study had been approved by the local ethics committee. All clinical investigation has been conducted according to the principles expressed in the Declaration of Helsinki.

### Study Subjects

We reviewed our consecutively collected acute anterior LAO patients with CT perfusion (CTP) scans within 6 h after stroke onset from March 2018 to May 2019. Patients were enrolled if they had (1) middle cerebral artery M1 segment and/or intracranial internal carotid artery (ICA) occlusion on pretreatment 4D-CTA reconstructed from CTP and (2) prestroke modified Rankin Scale score (mRS) ≤2. Patients were excluded if they (1) had bilateral acute ischemic lesions, (2) had poor image quality due to motion artifact, and (3) could not tolerate follow-up CT before discharge.

### Reperfusion Therapy

Patients who arrived at our center within 4.5 h of symptom onset with no hemorrhage or significant low-density lesion on baseline non-contrast CT (NCCT) images received alteplase at a dose of 0.9 mg/kg (maximum dose = 90 mg). Patients exhibiting no clinical improvement after thrombolysis and patients who arrived at 4.5–6 h after stroke onset were thrombectomy candidates. Thrombectomy was performed as bridging therapy after intravenous thrombolysis or as primary therapy in accordance with the guidelines of the time. Eligible patients were assigned to thrombectomy or non-thrombectomy groups based on whether their families consented to the procedure. The retrieval devices for thrombectomy were all Solitaire AB (ev3, Covidian, Dublin, Ireland). The detailed surgical procedure was according to EXTEND-IA trial (7).

### Imaging Protocols

All patients underwent baseline NCCT and CTP and follow-up NCCT at 24–48 h after admission. All CT imaging was acquired on a 320-detector row 640-slice cone beam MDCT scanner (Aquilion One, Toshiba Medical Systems). Whole-brain NCCT was performed in one rotation (detector width, 16 cm). Subsequent to NCCT, a CTP was acquired after administration of 50 ml of contrast agent (Ultravist 370; Bayer HealthCare, Berlin, Germany) injected intravenously at a rate of 6 ml/s chased by 50 ml of saline (acquisition parameters: 120 kV, 128 mAs; scanning coverage = 240 mm, scanning width = 5 mm). Starting 7 s after contrast injection, pulsed full rotation scan with 18 time points acquired over 60 s with a total pulse image acquisition time of 9.5 s was used. The scanning protocol of follow-up NCCT was the same as that of baseline NCCT.

### Imaging Analysis

Two raters (SZ and ZW) who jointly evaluated the imaging markers (SMCV, PH, and midline shift) were blinded to the patients' 24-h imaging and clinical data. A single trained observer (SZ) measured imaging markers in all patients twice, at an interval of 1 month apart. Another observer (ZW) independently made the same evaluation.

### Absent Filling of Ipsilateral SMCV (SMCV–)

We assessed the SMCV on 4D-CTA reconstructed from CTP by Vitrea fX (Version 1.0, Vital images, Minnetonka, MN, USA). Images were analyzed using maximum intensity projection (MIP). SMCV was defined as negative (SMCV–) if no contrast filling of SMCV was observed across the whole venous phase in the ischemic hemisphere, while the presence of contrast filling of ipsilateral SMCV at any time point of venous phase was marked as SMCV+. Patients with only contralateral absent contrast filling of SMCV were also designated as SMCV+.

### Assessment of Hypoperfusion and Ischemic Core Volume

A threshold of delay time >3 s was used for volumetric measurement of baseline hypoperfusion area, and relative cerebral blood flow (rCBF) <30% was used for calculating ischemic core volume (8).

### Neurological Outcome Measures

Midline shift was defined as the displacement of the septum pellucidum (or cavum septi pellucidi) more than 3 mm from the spatial midline on 24-h follow-up NCCT (9). According to this definition, patients were divided into two groups: midline shift and non-midline shift.

Hemorrhagic transformation was classified as hemorrhagic infarction (HI) or parenchymal hemorrhage (PH), according to the European Cooperative Acute Stroke Study (ECASS) definition. Symptomatic intracranial hemorrhage (sICH) was defined as any intracranial hemorrhage associated with an increase of ≥4 points on National Institutes of Health Stroke Scale (NIHSS) or death (10).

Reperfusion status was assessed on digital subtraction angiograms (DSA) using modified thrombolysis in cerebral infarction (mTICI) scores (11) in patients who received thrombectomy. We defined an mTICI score of 0–2a as no reperfusion and a score of 2b−3 as successful reperfusion.

NIHSS score was evaluated at 3 time points: baseline, 24–72 h after admission and discharge. Modified Rankin Scale (mRS) score was used to identify the clinical outcome at discharge. mRS score of 0–3 was designated a good outcome, and mRS score of 4–6 was designated a poor outcome. Death was defined as the outcome in patients who were dead (mRS scored 6) at discharge or died within 24 h after discharge.

### Statistical Analysis

Kappa statistics was used to test inter- and intrarater reliability for detecting the presence of SMCV–, poor collaterals, PH, and midline shift. Excellent inter- and intraobserver reliability was seen in distinguishing the SMCV– (κ = 0.869 and 0.870), poor collaterals (κ = 0.804 and 0.916), midline shift (κ = 0.818 and 0.912), and PH (κ = 0.863 and 0.929).

All numeric variables are expressed as mean ± SD or median (interquartile range, IQR). Categorical variables are presented as frequency (percentage). Fisher's exact test was used to compare dichotomous variables between groups, while Mann–Whitney *U*-test was used for ordered categorical variables, and independent samples two-tailed *t-*test or Mann–Whitney *U-*test was used for continuous variables, depending on the normality of the distribution. Variables identified by univariate analysis (*P* < 0.05) were included in multivariate regression model. All analyses were performed blinded to the participant identifying information. Receiver operating characteristic (ROC) analysis was performed to assess the performance of baseline characteristics of interest in predicting midline shift. The sensitivity and specificity were identified at the levels that maximized Youden value. Statistical analysis was performed using SPSS 18 (SPSS Inc., Chicago, IL, USA). ROC curve analyses were conducted by Medcalc statistical software version 15 (Medcalc Software, Mariakerke, Belgium). *P* < 0.05 was considered statistically significant.

## Results

### Overall Characteristics

**Figure 1:**
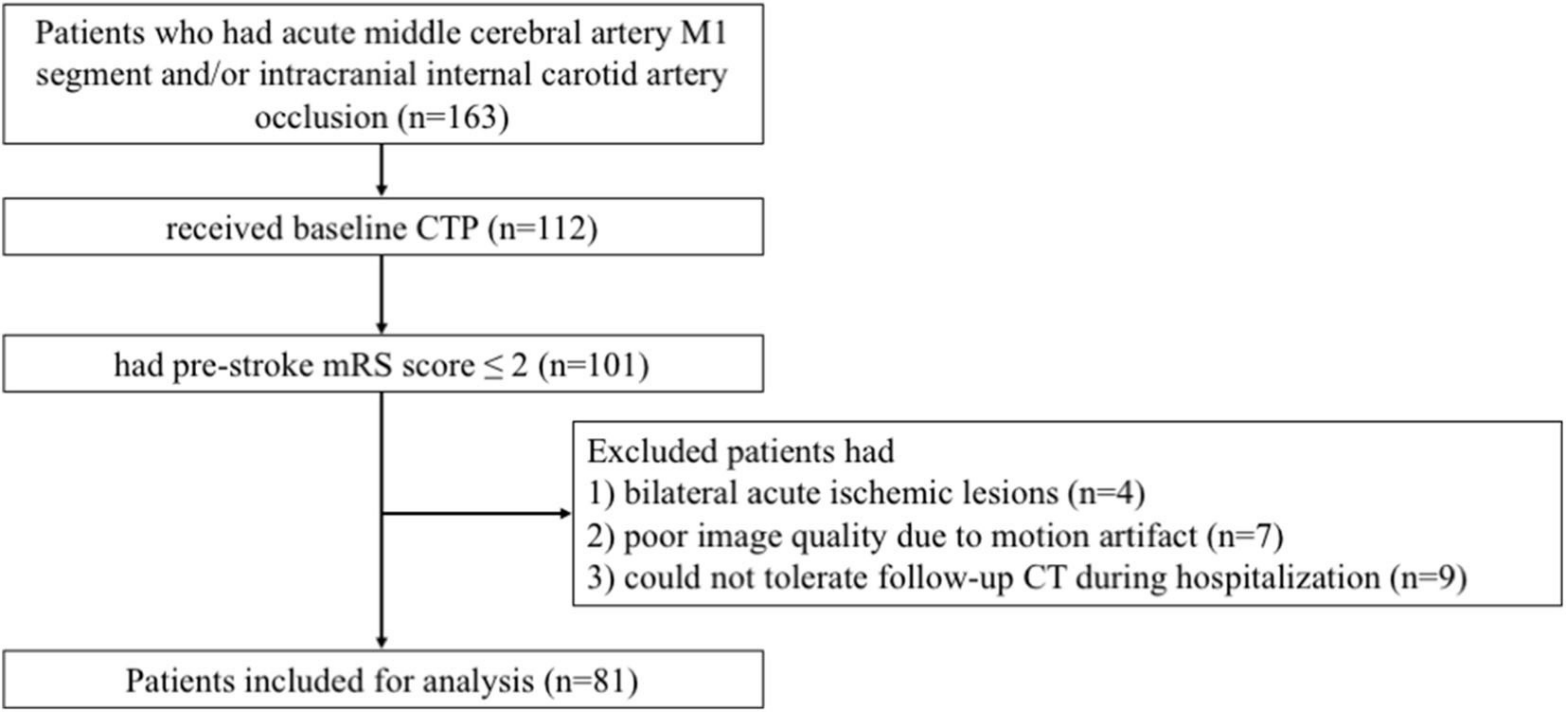
Flow chart of patients screening.

A total of 81 patients were enrolled in the analysis with mean age of 70.2 ± 14.5 years and median baseline NIHSS score of 20 (IQR, 13–23; Figure 1). Of 66 patients (81.5%) who received reperfusion therapies, 52 received thrombectomy (including 17 patients who received thrombolysis bridging with thrombectomy) and 84.6% achieved successful reperfusion through thrombectomy. Twenty-four (29.6%) patients had midline shift within 48 h after stroke. Notably, no patient with midline shift had a good outcome at discharge, and 14 (58.3%) were dead at discharge or within 24 h after discharge, a significantly higher proportion compared to non-midline shift patients (all *P* < 0.05). Baseline characteristics, radiological parameters, and outcome characteristics of all 81 patients are described in Supplementary Tables 1, 2.

### The Association Between Midline Shift and SMCV–

SMCV– was detected in 31 patients (38.3%). Univariate analysis showed that the presence of SMCV– was associated with a higher baseline NIHSS score, a larger volume of baseline hypoperfusion and ischemic core volume, and a higher rate of midline shift (*P* < 0.05) (Supplementary Table 3). Compared with non-midline shift group (n = 57), midline shift group had a higher baseline NIHSS score, a larger volume of hypoperfusion and ischemic core volume, and higher rate of SMCV– (*P* < 0.05; Supplementary Table 1).

Multivariate analysis showed that, after adjusting for baseline NIHSS score, both baseline ischemic core volume [odds ratio (OR) = 1.018, 95%CI = 1.002–1.034, *P* = 0.031] and SMCV– (OR = 14.328, 95%CI = 3.171–64.733, *P* = 0.001) were independently associated with the occurrence of midline shift.

When reperfusion therapies (thrombolysis and/or thrombectomy) were included as variables in the logistic regression model, they did not reduce the significance of SMCV– for midline shift (OR = 27.697, 95%CI = 5.608–136.801, *P* < 0.001), and reperfusion therapies were not associated with midline shift (all *P* > 0.05; Supplementary Table 4).

### The Comparison of Predictive Value Between Baseline Ischemic Core Volume and the Presence of SMCV–

ROC analysis showed that the area under the curve (AUC) of the model including only baseline ischemic core volume was 0.841 (95%CI = 0.743 – 0.939, *P* < 0.001), with a sensitivity of 79.2% and a specificity of 78.9% (Youden index, 0.581). AUC of the model including SMCV– was 0.850 (95%CI = 0.754 – 0.946, *P* < 0.001), with a sensitivity of 87.5% and a specificity of 82.5% (Youden index, 0.70). Comparisons of ROC curves between models using SMCV– and baseline ischemic core volume showed no significant difference (*Z* = 0.140, *P* = 0.889).

**Figure 2:**
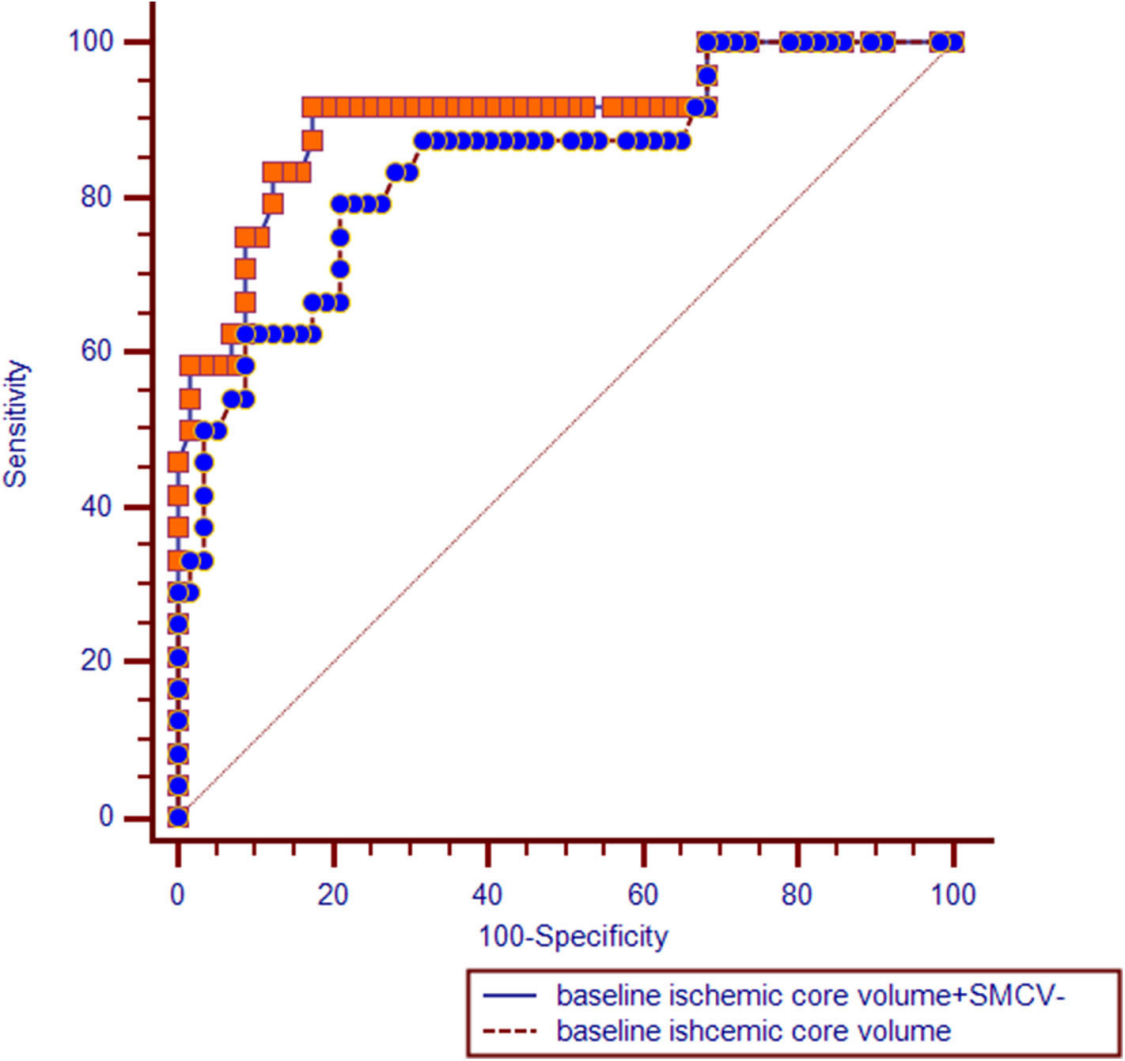
Receiver operating characteristic (ROC) curves comparisons. SMCV–, absent filling of superficial middle cerebral vein.

In multivariate regression analysis, the predictive value of the model including SMCV– and baseline ischemic core volume (AUC = 0.903, 95%CI = 0.822 – 0.984, *P* < 0.001) was significantly higher than the model with baseline ischemic core alone (Z = 2.451, *P* = 0.014; see Figure 2).

### Subgroup Analysis After Stratification of Contrast Filling of SMCV and Baseline Ischemic Core Volume

We further divided patients into four subgroups based on baseline ischemic core volume: 0–40 ml, 41–80 ml, 81–120 ml, and more than 120 ml.

In SMCV+ patients, we found no significant difference in the rate of midline shift between four groups (5.7 vs. 0% vs. 0 vs. 25%, χ^2^ = 0.745, *P* = 0.388). While in SMCV– patients, the rate of midline shift increased significantly with the increase in baseline ischemic core volume (25 vs. 54.5% vs. 66.7 vs. 92.3%, χ^2^ = 6.136, *P* = 0.013).

In subgroup comparisons, SMCV– patients showed a higher rate of midline shift than SMCV+ patients in subgroups of more than 80 ml (81–120 ml subgroup: 66.7 vs. 0%, χ^2^ = 5.833, *P* = 0.016; more than 120 ml subgroup: 92.3 vs. 25%, χ^2^ = 7.702, *P* = 0.006; Supplementary Table 5).

**Figure 3:**
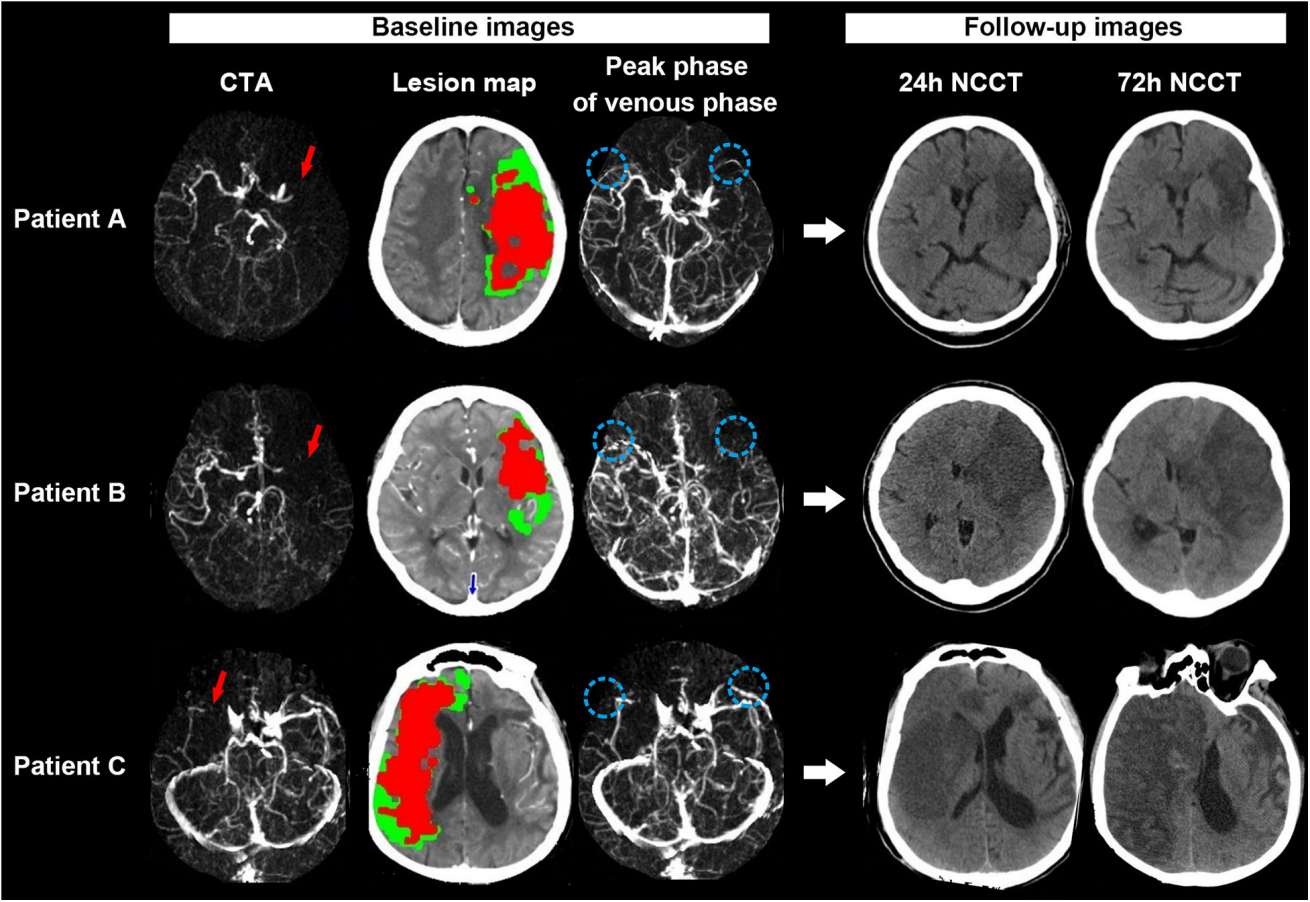
Examples for illustrating the associations between SMCV–, baseline ischemic core volume, and midline shift. Patient A: A 67-year-old woman with acute left middle cerebral artery occlusion (MCAO) [red long arrow on CT angiography (CTA)] presented with 107 ml baseline ischemic core volume (red color area on the lesion map) but symmetric SMCV (SMCV+) (blue circle on venous phase map) on 4-dimensional computed tomographic angiography (4D-CTA). She refused to accept thrombectomy after admission. On 24 h noncontrast CT (NCCT) after admission, we did not find significant brain edema on his follow-up NCCT scans. Her National Institutes of Health Stroke Scale (NIHSS) score and modified Rankin scale (mRS) score at discharge were 18 and 4, respectively. Patient B: A 70-year-old woman with acute left MCAO (red long arrow) presented with 61 ml baseline ischemic core volume (red color area on the lesion map) but the absence of ipsilateral SMCV (SMCV–) (blue circle on venous phase map) on 4D-CTA. She received thrombectomy at 6 h from stroke onset but failed to achieve successful reperfusion (modified TICI scored 0). Midline shift was observed in her 24 h NCCT and progressed gradually. She refused to accept decompressive craniectomy. Her NIHSS score and mRS score at discharge were 28 and 5, respectively. Patient C: A 87-year-old man with acute right MCAO (red long arrow) presented with both large ischemic core (158 ml: red color area on the lesion map) and SMCV– (blue circle on venous phase map) on 4D-CTA at baseline. He refused to receive thrombectomy or decompressive craniectomy after admission. His 24 and 72 h NCCT showed significant midline shift. His NIHSS score and mRS score at discharge were 37 and 5, respectively.

Figure 3 shows examples of the association of baseline ischemic core volume and SMCV– with midline shift.

## Discussion

In acute anterior LAO patients, the presence of SMCV– was strongly associated with the occurrence of midline shift, with a sensitivity of 87.5% and a specificity of 82.5%, and it increased the predictive value of baseline ischemic core volume for midline shift.

We found that SMCV– was significantly associated with the occurrence of midline shift in acute LAO. Injury of the SMCV (also known as the Sylvian vein) has been reported to correlate with the progress of brain edema in pterion approach surgery (5). While in ischemic stroke affecting the Sylvian fissure, venules within the Sylvian fissure may be rapidly occluded by thrombosis after the initial vessel occlusion (12), so as to influence the opacification of SMCV on CTA. Due to venule occlusions, the resistance to cerebrospinal fluid absorption will increase, resulting in the elevation of venous pressure. This may increase fluid leakage into the perivascular space resulting in brain edema, even to the point of midline shift (13). In our study, the rate of SMCV– in LAO patients was 38.4%, higher than the rate (22.4%) in our previous study of thrombolytic patients, supporting the hypothesis that the extent of ischemia may influence the occurrence of SMCV–.

Pretreatment evaluation of contrast filling of the SMCV has clinical significance. In our study, we demonstrated that SMCV– was comparable to baseline ischemic core volume in predicting midline shift, and the joint use of SMCV– and baseline ischemic core volume can better predict midline shift, especially seen in patients with large ischemic core (over 80 ml). It is widely known that infarct volume is a commonly used surrogate of brain edema. The 2014 American Heart Association (AHA)/American Stroke Association (ASA) guideline for the management of cerebral edema after stroke recommended that the volume measurement on diffusion-weighted imaging (DWI) within 6 h is useful, and volumes over 80 ml predict rapid fulminant course (14). However, DWI has limited availability in emergency settings and restricted feasibility in severely ill stroke patients. While CTP is more practical for acute stroke imaging, assessment of infarct volume in this modality strongly relies on commercial reconstruction software. Compared to the assessment of ischemic core volume, the identification of SMCV– depends on CTP-derived 4D-CTA, which not only can be realized in MIStar but is also accessible in most CT workstations using widely available volume rendering technique (VRT) reconstruction software, such as Vitrea fX, Inspace, etc. Therefore, the pretreatment evaluation of SMCV– in stroke patients can be widely applied. Furthermore, in centers with advanced imaging postprocessing software, joint evaluation of both ischemic core volume and contrast filling of SMCV before treatment can provide a more accurate prediction of midline shift than that of ischemic core volume alone, which can help us screen patients at high risk of cerebral hernia early.

This study had limitations. First, this is a small sample and retrospective study. Second, long-term follow-up data were not included in this study, as many patients suffered rapid neurological deterioration acute LAO, which often led to death within several days after stroke onset. Therefore, we focused on observing the early clinical outcomes in our acute LAO patients in this study. Third, as the follow-up CT perfusion was not a regular test for our patients, and some patients who did not receive thrombectomy but had severe neurological deficits were unable to stand follow-up CT perfusion examination, we were unable to evaluate their reperfusion status. Therefore, we did not assess the effect of reperfusion on the presence of SMCV– and the association between SMCV– and midline shift. However, we included reperfusion therapies as variables in our analysis, which can partially adjust for the impact of reperfusion therapy on the association between SMCV– and midline shift.

In conclusion, the presence of SMCV– in acute anterior LAO patients could predict the formation of midline shift and supplement the predictive value of baseline ischemic core for midline shift. Therefore, SMCV– may be a new ultra-early target for future clinical prevention of midline shift.

## Data Availability Statement

The raw data supporting the conclusions of this article will be made available by the authors, without undue reservation.

## Ethics Statement

The studies involving human participants were reviewed and approved by the ethics committee of Zhejiang Provincial People's Hospital. The patients/participants provided their written informed consent to participate in this study.

## Author Contributions

SZ, LL, MP, and YG contributed to the conception and design of the study. SZ, LL, RZ, and ZS contributed to the acquisition and analysis of the data. SZ, LL, and YY contributed to drafting the text and SZ, RZ, and MW in preparing the figures. All authors contributed to the article and approved the submitted version.

## Conflict of Interest

The authors declare that the research was conducted in the absence of any commercial or financial relationships that could be construed as a potential conflict of interest.

## References

[1] Huang X Yang Q Shi X Xu X Ge L Ding X. Predictors of malignant brain edema after mechanical thrombectomy for acute ischemic stroke. J Neurointerv Surg. (2019) 11:994–8. 10.1136/neurintsurg-2018-014650 30798266

[2] Hofmeijer J Kappelle LJ Algra A Amelink GJ van Gijn J van der Worp HB. Surgical decompression for space-occupying cerebral infarction (the hemicraniectomy after middle cerebral artery infarction with life-threatening edema trial [hamlet]): a multicentre, open, randomised trial. Lancet Neurol. (2009) 8:326–33. 10.1016/S1474-4422(09)70047-X 19269254

[3] Jo K Bajgur SS Kim H Choi HA Huh PW Lee K. A simple prediction score system for malignant brain edema progression in large hemispheric infarction. PLoS One. (2017) 12:e0171425. 10.1371/journal.pone.0171425 28178299 PMC5298259

[4] Horsch AD Dankbaar JW Stemerdink TA Bennink E van Seeters T Kappelle LJ. Imaging findings associated with space-occupying edema in patients with large middle cerebral artery infarcts. AJNR Am J Neuroradiol. (2016) 37:831–7. 10.3174/ajnr.A4637 26797136 PMC7960291

[5] Dean BL Wallace RC Zabramski JM Pitt AM Bird CR Spetzler RF. Incidence of superficial sylvian vein compromise and postoperative effects on ct imaging after surgical clipping of middle cerebral artery aneurysms. AJNR Am J Neuroradiol. (2005) 26:2019–26. 16155152 PMC8148832

[6] Zhang S Lai Y Ding X Parsons M Zhang JH Lou M. Absent filling of ipsilateral superficial middle cerebral vein is associated with poor outcome after reperfusion therapy. Stroke. (2017) 48:907–14. 10.1161/STROKEAHA.116.016174 28265013 PMC5374052

[7] Campbell BCV Mitchell PJ Yan B Parsons MW Christensen S Churilov L. A multicenter, randomized, controlled study to investigate EXtending the time for Thrombolysis in Emergency Neurological Deficits with Intra-Arterial therapy (EXTEND-IA). Int J Stroke. (2014) 9:126–32. 10.1111/ijs.12206 24207098

[8] Bivard A Levi C Spratt N Parsons M. Perfusion CT in acute stroke: a comprehensive analysis of infarct and penumbra. Radiology. (2013) 267:543–50. 10.1148/radiol.12120971 23264345

[9] Gibson JY Massingale TW Graves GR LeBlanc MH Meydrech EF. Relationship of cranial midline shift to outcome of very-lowbirth-weight infants with periventricular hemorrhagic infarction. J Neuroimaging. (1994) 4:212–7. 10.1111/jon199444212 7949559

[10] Larrue V von Kummer R Müller A Bluhmki E. Risk factors for severe hemorrhagic transformation in ischemic stroke patients treated with recombinant tissue plasminogen activator: a secondary analysis of the European-Australasian Acute Stroke Study (ECASS II). Stroke. (2001) 32:438–41. 10.1161/01.STR.32.2.438 11157179

[11] Dargazanli C Consoli A Barral M. Impact of modified tici 3 versus modified tici 2b reperfusion score to predict good outcome following endovascular therapy. AJNR Am J Neuroraiol. (2017) 38:90–96. 10.3174/ajnr.A4968 27811134 PMC7963649

[12] Sasaki M Honmou O Radtke C Kocsis JD. Development of a middle cerebral artery occlusion model in the nonhuman primate and a safety study of i.V. Infusion of human mesenchymal stem cells. PLoS One. (2011) 6:e26577. 10.1371/journal.pone.0026577 22039510 PMC3200343

[13] Alperin N Lee SH Mazda M Hushek SG Roitberg B Goddwin J. Evidence for the importance of extracranial venous flow in patients with idiopathic intracranial hypertension (iih). Acta Neurochir Suppl. (2005) 95:129–32 10.1007/3-211-32318-X_28 16463836

[14] Powers WJ Rabinstein AA Ackerson T Adeoye OM Bambakidis NC Becker K. Guidelines for the early management of patients with acute ischemic stroke: 2019 update to the 2018 guidelines for the early management of acute ischemic stroke: a guideline for healthcare professionals from the American Heart Association/American Stroke Association. Stroke. (2019) 50:e344–e418. 10.1161/STR.0000000000000211 31662037

