# Supplementary material for: Absent Contrast Filling of Ipsilateral Superficial Middle Cerebral Vein Predicts Midline Shift in Acute Middle Cerebral Artery Occlusion
Source: Front Neurol. 2020 Nov 5;11:570844. doi: 10.3389/fneur.2020.570844 (PMC7674643; doi:10.3389/fneur.2020.570844)
Supplement: Supplementary file 1 [file Data_Sheet_1.docx]

Supplementary Material

# Supplementary Tables

**Supplementary Table I Baseline demographics and imaging variables, stratified by non-midline shift vs midline shift**

| **Characteristics** | **All**  **(n=81)** | **Non-midline shift (n=57)** | **Midline shift**  **(n=24)** | **Test value** | ***P* value** |
| --- | --- | --- | --- | --- | --- |
| Female, n (%) | 27 (33.3) | 21 (36.8) | 6 (25.0) | χ^2^=1.066 | 0.302 |
| Age (year), mean±SD | 70.2±14.5 | 69.6±15.5 | 71.6±12.1 | t=-0.560 | 0.577 |
| Transferred from local hospitals, n (%) | 40 (49.4) | 27 (47.4) | 13 (54.2) | χ^2^=0.312 | 0.576 |
| OIT (min), mean±SD | 234.2±128.1 | 230.7±125.1 | 242.3±137.3 | t=-0.366 | 0.715 |
| Baseline NIHSS score, median (IQR) | 20 (13-23) | 17 (12-22) | 22 (19-26) | Z=-3.351 | 0.001 |
| Hypertension, n (%) | 55 (67.9) | 42 (73.7) | 13 (54.2) | χ^2^=2.952 | 0.086 |
| Diabetes mellitus, n (%) | 15 (18.5) | 12 (21.1) | 3 (12.5) | χ^2^=0.819 | 0.366 |
| Atrial fibrillation, n (%) | 29 (35.8) | 19 (33.3) | 10 (41.7) | χ^2^=0.510 | 0.475 |
| Previous stroke, n (%) | 18 (22.2) | 10 (17.5) | 8 (33.3) | χ^2^=2.436 | 0.119 |
| Coronary artery disease, n (%) | 14 (17.3) | 10 (17.5) | 4 (16.7) | χ^2^=0.009 | 0.924 |
| Temperature (℃), mean±SD | 36.8±0.4 | 36.8±0.5 | 36.7±0.3 | t=0.995 | 0.323 |
| Baseline systolic blood pressure (mmHg), mean±SD | 149.5±25.1 | 152.6±27.1 | 142.0±18.0 | t=1.274 | 0.206 |
| Baseline diastolic blood pressure (mmHg), mean±SD | 81.0±13.7 | 81.5±15.0 | 79.8±10.3 | t=-0.495 | 0.622 |
| Baseline serum glucose (mmol/L), mean±SD | 7.7±2.7 | 7.7±2.7 | 7.8±2.9 | t=-0.083 | 0.934 |
| Baseline hypoperfusion volume (ml), mean±SD | 183.8±102.1 | 157.1±83.5 | 247.3±115.5 | t=-3.950 | <0.001 |
| Baseline ischemic core volume (ml), mean±SD | 70.8±75.4 | 43.4±43.9 | 135.9±93.8 | t=-6.073 | <0.001 |
| SMCV-, n (%) | 31 (38.3) | 10 (17.5) | 21 (87.5) | χ^2^=34.986 | <0.001 |
| Reperfusion therapies, n (%) |  |  |  |  |  |
| Intravenous thrombolysis | 31 (38.3) | 24 (42.1) | 7 (29.2) | χ^2^=1.197 | 0.274 |
| Thrombectomy | 52 (64.2) | 38 (66.7) | 14 (58.3) | χ^2^=0.510 | 0.475 |
| Bridging therapy | 17 (21.0) | 14 (24.6) | 3 (12.5) | χ^2^=1.482 | 0.224 |

SD, standard deviation; IQR, interquartile range; OIT: onset to imaging time; NIHSS, National Institute of Health Stroke Scale; SMCV-, absent filling of ipsilateral superficial middle cerebral vein; Bridging therapy, thrombolysis bridging with thrombectomy.

**Supplementary Table II Clinical outcome stratified by non-midline shift vs midline shift**

| **Characteristics** | **All**  **(n=81)** | **Non-midline shift (n=57)** | **Midline shift**  **(n=24)** | **Test value** | ***P* value** |
| --- | --- | --- | --- | --- | --- |
| NIHSS score at 24-48 hours, median (IQR) | 18 (8-35) | 13 (5.5-21) | 37 (24-37) | Z=-5.156 | <0.001 |
| NIHSS score at discharge, median (IQR) | 14 (6-26) | 9 (4-17) | 34 (21-37) | Z=-5.461 | <0.001 |
| HT, n (%) | 36 (44.4) | 21 (36.8) | 15 (62.5) | χ^2^=4.503 | 0.034 |
| PH, n (%) | 18 (22.2) | 7 (12.3) | 11 (45.8) | χ^2^=11.000 | 0.001 |
| sHT, n (%) | 8 (9.9) | 2 (3.5) | 6 (25.0) | χ^2^=8.764 | 0.003 |
| Decompressive craniectomy, n (%) | 13 (16.0) | 2 (3.5) | 11 (45.8) | χ^2^=12.455 | <0.001 |
| Poor outcome, n (%) | 60 (74.1) | 36 (63.2) | 24 (100.0) | 11.937 | 0.001 |
| Death, n (%) | 22 (27.2) | 8 (14.0) | 14 (58.3) | χ^2^=16.752 | <0.001 |

IQR, interquartile range; NIHSS, National Institute of Health Stroke Scale; HT, hemorrhagic transformation; PH, parenchymal hemorrhage; sHT, symptomatic hemorrhagic transformation.

Supplementary table III. Clinical and imaging characteristics that stratified by SMCV+ vs SMCV-

| Characteristics | SMCV+  n=50 | SMCV-  n=31 | Test value | *P* value |
| --- | --- | --- | --- | --- |
| Female, n (%) | 18 (36.0) | 9 (29.0) | χ^2^=0.418 | 0.518 |
| Age (year), mean±SD | 70.9±13.3 | 69.1±16.4 | t=0.529 | 0.598 |
| Transferred from local hospitals, n (%) | 21 (42.0) | 19 (61.3) | χ^2^=2.849 | 0.091 |
| Reperfusion therapies, n (%) |  |  |  |  |
| Intravenous thrombolysis | 21 (42.0) | 10 (32.3) | χ^2^=0.769 | 0.381 |
| Thrombectomy | 31 (62.0) | 21 (67.7) | χ^2^=0.274 | 0.600 |
| Bridging therapy | 11 (22.0) | 6 (19.4) | χ^2^=0.081 | 0.776 |
| OIT (min), mean±SD | 230.3±127.2 | 240.3±131.6 | t=-0.335 | 0.738 |
| Baseline NIHSS score, median (IQR) | 17 (8-25) | 20 (16-23) | t=-4.152 | <0.001 |
| Hypertension, n (%) | 36 (72.0) | 19 (61.3) | χ^2^=1.007 | 0.316 |
| Diabetes mellitus, n (%) | 10 (20.0) | 5 (16.1) | χ^2^=0.190 | 0.663 |
| Atrial fibrillation, n (%) | 17 (34.0) | 12 (38.7) | χ^2^=0.185 | 0.667 |
| Previous stroke, n (%) | 9 (18.0) | 9 (29.0) | χ^2^=1.348 | 0.246 |
| Coronary artery disease, n (%) | 7 (14.0) | 7 (22.6) | χ^2^=0.985 | 0.321 |
| Temperature (℃), mean±SD | 36.8±0.4 | 36.8±0.5 | t=0.317 | 0.752 |
| Baseline systolic blood pressure (mmHg), mean±SD | 147.4±23.8 | 152.9±27.1 | t=-0.956 | 0.342 |
| Baseline diastolic blood pressure (mmHg), mean±SD | 79.4±14.1 | 83.5±12.9 | t=-1.307 | 0.195 |
| Baseline serum glucose (mmol/L) , mean±SD | 7.8±2.8 | 7.5±2.7 | t=0.471 | 0.639 |
| Baseline hypoperfusion volume (ml), mean±SD | 151.5±85.0 | 235.9±107.1 | t=-3.928 | <0.001 |
| Baseline ischemic core volume (ml), mean±SD | 47.8±78.0 | 123.6±5.6 | t=-4.094 | <0.001 |
| NIHSS score at 24-48 hours, median (IQR) | 12.5 (5-21) | 32 (20-37) | Z=-4.706 | <0.001 |
| NIHSS score at discharge, median (IQR) | 7.5 (3-18) | 25 (16-37) | Z=-5.013 | <0.001 |
| HT, n (%) | 20 (40.0) | 16 (51.6) | χ^2^=1.045 | 0.307 |
| PH, n (%) | 8 (16.0) | 10 (32.3) | χ^2^=2.926 | 0.105 |
| sHT, n (%) | 4 (8.0) | 4 (12.9) | χ^2^=0.517 | 0.472 |
| Midline shift, n (%) | 3 (6.0) | 21 (67.7) | χ^2^=34.986 | <0.001 |
| Decompressive craniectomy, n (%) | 1 (2.0) | 12 (38.7) | χ^2^=19.139 | <0.001 |
| Poor outcome, n (%) | 29 (58.0) | 31 (100.0) | χ^2^=17.577 | <0.001 |
| Death, n (%) | 7 (14.0) | 15 (48.4) | χ^2^=11.438 | 0.001 |

SD, standard deviation; IQR, interquartile range; Bridging therapy, thrombolysis bridging with thrombectomy; OIT: onset to imaging time; NIHSS, National Institute of Health Stroke Scale; SMCV-, absent filling of ipsilateral superficial middle cerebral vein; HT, hemorrhagic transformation; PH, parenchymal hemorrhage; sHT, symptomatic hemorrhagic transformation.

Supplementary table IV. Binary logistic regression analysis for midline shift

|  | OR | 95%CI | *P* value |
| --- | --- | --- | --- |
| SMCV- | 27.697 | 5.608-136.801 | <0.001 |
| No reperfusion therapy | - | - | 0.499 |
| Thrombolysis | 0.904 | 0.075-10.906 | 0.937 |
| Thrombectomy | 0.475 | 0.064-3.524 | 0.467 |
| Bridging therapy | 0.189 | 0.018-2.015 | 0.168 |
| Baseline NIHSS | 1.026 | 0.906-1.162 | 0.688 |
| Baseline core volume | 1.006 | 0.998-1.014 | 0.122 |

SMCV, superficial middle cerebral vein; NIHSS, National Institute of Health Stroke Scale; Bridging therapy, thrombolysis combined with thrombectomy.

Supplementary table V. Subgroup comparisons for the rate of midline shift

| Baseline ischemic core volume categories | SMCV+ (n=50) | SMCV- (n=31) | Test value | P value |
| --- | --- | --- | --- | --- |
| 0-40ml (n=39) | 2 (5.7) | 1 (25.0) | χ^2^=1.880 | 0.170 |
| 41-80ml (n=10) | 0 (0) | 6 (54.5) | χ^2^=3.636 | 0.057 |
| 81-120ml (n=15) | 0 (0) | 2 (66.7) | χ^2^=5.833 | 0.016 |
| More than 120ml (n=17) | 1 (25.0) | 12 (92.3) | χ^2^=7.702 | 0.006 |

SMCV, superficial middle cerebral vein.
